# A Novel Enzyme-Free Ratiometric Fluorescence Immunoassay Based on Silver Nanoparticles for the Detection of Dibutyl Phthalate from Environmental Waters

**DOI:** 10.3390/bios12020125

**Published:** 2022-02-16

**Authors:** Hui Meng, Nannan Yao, Kun Zeng, Nuanfei Zhu, Yue Wang, Biying Zhao, Zhen Zhang

**Affiliations:** 1 School of the Environment and Safety Engineering, Jiangsu University, Zhenjiang 212013, China; menghui@ujs.edu.cn (H.M.); 2211909008@stmail.ujs.edu.cn (N.Y.); kjj80116@ujs.edu.cn (K.Z.); zhunuanfei@163.com (N.Z.); wangyue_persnl@126.com (Y.W.); 2 International Genome Center, Jiangsu University, Zhenjiang 212013, China; zhaoby@ujs.edu.cn

**Keywords:** immunoassay, antibody, dibutyl phthalate, scopoletin, amplex red, silver nanoparticles

## Abstract

A novel ratiometric fluorescent immunoassay was developed based on silver nanoparticles (AgNPs) for the sensitive determination of dibutyl phthalate (DBP). In the detection system, AgNPs were labeled on the secondary antibody (AgNPs@Ab_2_) for signal amplification, which aimed to regulate the H_2_O_2_ concentrations. When AgNPs-Ab_2_ and antigen–primary antibody (Ab_1_) were linked by specific recognition, the blue fluorescence of Scopoletin (SC) could be effectively quenched by the H_2_O_2_ added while the red fluorescence of Amplex Red (AR) was generated. Under the optimized conditions, the calculated detection of limit (LOD, 90% inhibition) reached 0.86 ng/mL with a wide linear range of 2.31–66.84 ng/mL, which was approximately eleven times lower than that by HRP-based traditional ELISA with the same antibody. Meanwhile, it could improve the inherent built-in rectification to the environment by the combination of the dual-output ratiometric fluorescence assays with ELISA, which also enhanced the accuracy and precision (recoveries, 87.20–106.62%; CV, 2.57–6.54%), indicating it can be applied to investigate the concentration of DBP in water samples.

## 1. Introduction

Dibutyl phthalate (DBP), a type of phthalate ester (PAE), is widely applied in the industry, medicine, agriculture, and domestication [1,2,3]. As it does not react chemically with the molecular bonds of polymer matrix, DBP can be easily released into the environment and has become a ubiquitous environmental contaminant. Previous studies have indicated that DBP can interfere with the normal hormone secretion of the human body, and has reproductive, embryological, and genetic toxicity even at trace level [4,5]. Considering its biological accumulation and slow biodegradation in the organization [1], developing efficient methods to detect DBP for environmental risk assessment is urgently needed. 

Nowadays, instrumental methods are commonly used to detect DBP [6,7,8]. These methods suffer from high cost, low throughput, and tedious sample pretreatment not to mention requiring advanced instruments and professional operation. Hence, these approaches are unsuitable for field studies and on-line monitoring of DBP [6]. By contrast, the enzyme-linked immunosorbent assay (ELISA) has become the most widely used analysis techniques in many fields, such as diseases diagnosis [9,10], food safety [11,12], and environmental monitoring [13,14]. It shows obvious advantages owing to its low cost, good specificity, automation, and high-throughput [11]. However, the traditional colorimetric ELISA which mainly relies on natural enzymes biocatalyzing a chromogenic substrate to colored molecules as signal output often suffers from low signal outputs, formidable background, and thus relatively low sensitivity and selectivity [15]. Considering that, a more sensitive and accurate immunosensor is urgently needed to fulfill the requirement for DBP monitoring.

Currently, many strategies, such as chemiluminescent ELISA [16], plasmonic ELISA [17], electrochemical ELISA [18] and fluorescence ELISA [19], have been developed to overcome the main drawbacks of the traditional colorimetric ELISA. Among this research, ratiometric fluorescence ELISA is a promising strategy for monitoring DBP in complex environmental matrices due to its superior properties such as high sensitivity and satisfactory accuracy, which are based on the ratio of the intensities of two different emission peaks as a signal [20,21] that not only reduces external interference but also improves detection sensitivity through self-calibration [22]. To improve the sensitivity and selectivity of the ratiometric fluorescence sensor, many attempts have been made using nanoparticles as an alternative to the nature enzyme or fluorescent labels. Zhu et al. [23] designed a novel ratiometric fluorescence ELISA based on a catalytic oxidation of OPD using as-prepared MnO_2_NF-mediated signal tagging with the help of exogenous CDs for C-reactive protein (CRP) detection. Lin et al. [24] developed a ratiometric fluorescence enzyme-linked immunosorbent assay for sensitive detection of ethyl carbamate (EC) by introducing fluorescent silicon nanoparticles (SiNPs) into the chromogenic substrate system (o-phenylenediamine (OPD)/H_2_O_2_). Although these excellent works showed that ratiometric fluorescence sensors have a huge potential for trace detection, the advantage of enzyme-mediated signal amplification, which is important for high-throughput analysis, was not applied [25,26,27].

**Scheme 1:**
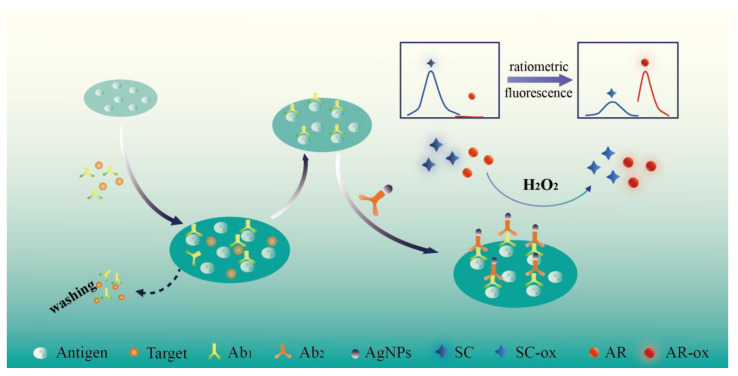
Schematic illustration of a simple ratiometric fluorescence ELISA for TBBPA determination based on AgNPs and H_2_O_2_.

In this study, a ratiometric fluorescence ELISA to detect DBP was developed. The principle of the sensing method is shown in Scheme 1. Firstly, silver nanoparticles (AgNPs) were prepared by a one-step method and applied in the ratiometric fluorescence ELISA system, which displayed excellent peroxidase-like activity toward the substrate Scopoletin (SC) and Amplex Red (AR). In addition, AgNPs were labeled on the secondary antibody (AgNPs@Ab_2_) to adjust the concentrations of H_2_O_2_, which was related to the magnitude of the signal response. After being optimized and evaluated, the proposed high-throughput method was applied to investigate the occurrence of DBP in a typical area.

## 2. Experiments and Materials

### 2.1. Synthesis of Ag Nanoparticles (AgNPs)

All the glass instruments used in the following procedure were repeatedly washed with ultra-pure water and dried in an oven at 37 °C. AgNPs were prepared by the published method with slight modifications [28]. Briefly, AgNO_3_ (0.1 g) was dissolved in 200 mL of deionized water and the solution transferred to a round-bottomed flask. When the solution was heated to a boil, 1% of sodium citrate (10 mL) was added slowly and heating was continued. AgNPs were synthesized successfully until the solution turned pale yellow. The obtained solution was stirred at 13,000 rpm for 15 min and the supernatant was also dialyzed for 3 days. Finally, the prepared solution was stored at 4 °C before use.

### 2.2. Synthesis of AgNPs@Ab_2_

The preparation of antibody marker AgNPs@Ab_2_ was based on the synthesis method of Zhang et al. [29], and the specific process was as follows: 1 mL of AgNP solution was put into a vial, to which 10 μL of sheep anti-rabbit solution (2 mg/mL) was added. The mixture was stirred at 37 °C for 12 h, then 500 μL 1.0 wt% BSA solution was added and stirring continued at a constant temperature. The resulting products were centrifuged, shaken, washed, redissolved in PBS solution, and stored at 4 °C for future use.

### 2.3. Catalytic Oxidation Assay

The catalytic activities of synthesized AgNPs were evaluated by fluorescence changes of SC and AR in the presence of H_2_O_2_. The specific experiment was carried out as follows: 200 μL of H_2_O_2_ solution (125 mM) was mixed with 10 μL of SC or AR in the absence or presence of AgNPs in a 500 μL centrifuge tube at 37 °C for 30 min. To ensure that SC and AR did not interfere with each other in the homogeneous system, we conducted the following experiments to verify: 200 μL of H_2_O_2_ solution (125 mM) was added into 20 μL of SC and AR with AgNPs or not in a 500 μL centrifuge tube at 37 °C for 30 min.

### 2.4. The Development of Ratiometric Fluorescence ELISA for DBP

First, DBP antigen (100 μL/well) was coated on the 96-well microplate at 4 °C using coating buffer overnight. After washing four times with PBS, each well was blocked with blocking buffer at 37 °C for 45 min. Then, 50 μL DBP antibody and 50 μL DBP were added to each well separately and kept at 37 °C for 30 min. Washing again, 100 μL of AgNPs-labeled secondary antibody was added into each well. After the steps of incubation and washing, 200 μL H_2_O_2_ and 20 μL SC and AR were added into each well at 37 °C for 30 min. Finally, the fluorescence intensity was measured by a microplate reader under the excitation wavelengths of 380 nm and 560 nm.

### 2.5. LC–MS/MS Analysis

To verify the reliability of this established method, samples of DBP in real water were determined by the LC–MS/MS method. The experiment details are described in the Supplementary Materials.

## 3. Result and Discussion

### 3.1. The Characterization of AgNPs and AgNPs@Ab_2_

**Figure 1:**
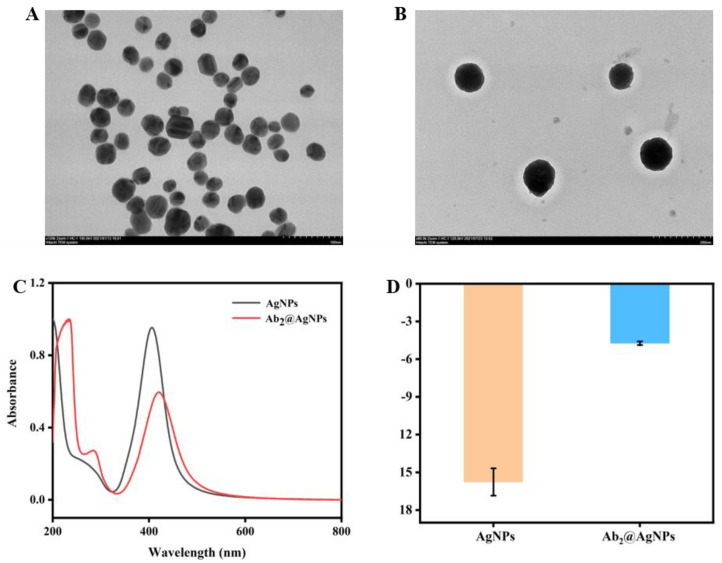
Characterization of AgNPs and Ab_2_@AgNPs. (**A**) Structure and TEM image of AgNPs, (**B**) structure and TEM image of Ab_2_@AgNPs, (**C**) The UV absorption spectra of AgNPs and AgNPs@Ab_2_, and (**D**) The Zeta potential analysis of AgNPs and AgNPs@Ab_2_.

Figure 1C shows that the ultraviolet absorption spectrum of bar AgNPs has a strong peak defined maximum close to 400 nm, consistent with the literature [30]. As shown in Figure 1A,B, we can see that AgNPs have a structure with a roughly spherical shape and a size of about 30 nm, which increased to 50 nm upon conjugation with Ab_2_. This increase was due to the agglomeration of particles that is often caused by the bonding between Ab_2_ and AgNPs [31]. As seen from Figure 1C, the ultraviolet absorption peak of AgNPs@Ab_2_ was obviously blue-shifted, indicating the successfully conjugations of AgNPs and Ab_2_ [29]. To further evaluate the synthesized AgNPs@Ab_2_, the Zeta potential values of AgNPs and AgNPs@Ab_2_ were measured to be −15.77 mV and −4.74 mV, respectively (Figure 1D). AgNPs@Ab_2_ was significantly lower than AgNPs, which was due to secondary antibody and silver nanoparticles being bound to each other by electrostatic adsorption, disrupting the homeostasis of the original colloid [32]. The result of the Zeta potential also proved the successful synthesis of AgNPs laterally.

### 3.2. The Peroxidase-like Activity of the Nanoparticles

**Figure 2:**
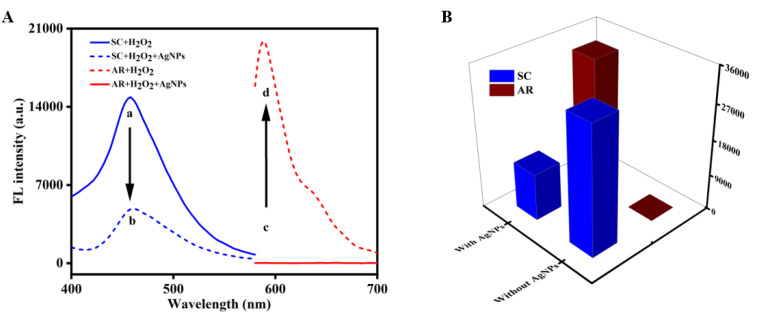
(**A**) Fluorescence spectra of separately used SC in (a) the absence of AgNPs, (b) the presence of AgNPs, and (c,d) separately used AR. (**B**) 3D fluorescent columns of simultaneously used SC (blue columns) and AR (red columns) without and with AgNPs.

**Figure 3:**
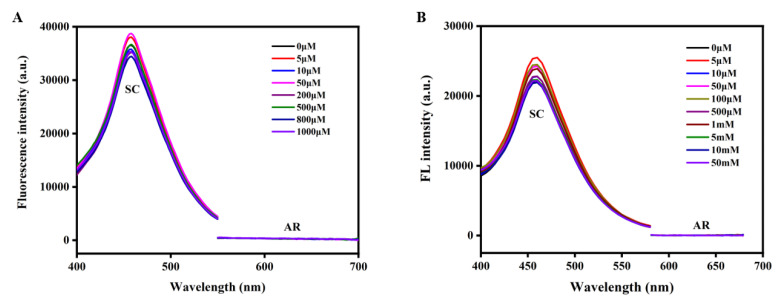
Fluorescence spectra of SC and AR under the different concentrations of Ag^+^ (**A**) and H_2_O_2_ (**B**).

As an important noble metal nanoparticle, AgNP has attracted extensive attention due to its low cost and high extinction coefficient [33]. Exploring the peroxidase-like properties of AgNPs can expand the application field of simulating the activity of nanoparticles [34,35]. To demonstrate the peroxidase activity of AgNPs, we tested the catalytic oxidation of SC and AR in the presence of H_2_O_2_, two fluorescent substrates that possess inverse responses. The emission peak of SC was at 465 nm, and produced almost non-fluorescent products (FI_465_, SC_-OX_) by oxidation. In contrast, AR was non-fluorescent, but could produce a significantly enhanced signal at 585 nm (FI_585_, AR_-OX_) after H_2_O_2_’s oxidation under the catalysis of peroxidase [36]. As shown in Figure 2A, SC presented a high fluorescent signal in the absence of H_2_O_2_ (curve a), but its fluorescence plummeted after the addition of AgNPs (curve b); AR had almost no fluorescence (curve c), while fluorescence was obviously enhanced at 585 nm (curve d). To ensure that Ag^+^ and H_2_O_2_ had no effect on substrate fluorescence, we verified this by measuring fluorescence at different concentrations of Ag^+^ and H_2_O_2_. Figure 3A,B indicates that Ag^+^ and H_2_O_2_ had no effect on the fluorescence signal. This was further illustrated that the change of fluorescence was caused by AgNPs. We also investigated whether the two substrates interfere with each other in a homogeneous system. As seen from Figure 2B, the 3D fluorescence columns of the two substrates in the absence or presence of AgNPs performed exactly the same as in their respective uses. These results proved that AgNPs have good oxidase-like properties.

### 3.3. Optimization of Experimental Conditions

In order to obtain better sensing performance, several of the parameters of immunoassay were optimized, including the concentrations of H_2_O_2_, AR, and SC, the reaction time of AgNPs@Ab_2_ and H_2_O_2_, and the pH. The fluorescence intensity ratio (F_585_/F_465_) was an important indicator for evaluating the performance of fluorescent immunoassay. Figure S1C indicates that with increasing H_2_O_2_, the fluorescence intensity ratio increased significantly until the concentration reached 125 mM. This was because 125 mM H_2_O_2_ can completely etch AgNPs, and beyond this level more H_2_O_2_ would not participate in the reaction. Therefore, 125 mM was selected as the optimal reaction concentration of H_2_O_2_ to achieve the best analytical performance. Additionally, Figure S1B also demonstrates that the most suitable reaction time between H_2_O_2_ and AgNPs was 30 min. Figure S1A reveals that with increasing of the pH value, F_585_/F_465_ initially increased and then decreased at pH 7, which was selected as an optimal value in the assay. At the same time, Figure S1D,E show that the optimal concentrations of AR and SC were 20 μM and 4 μM, respectively.

**Figure 4:**
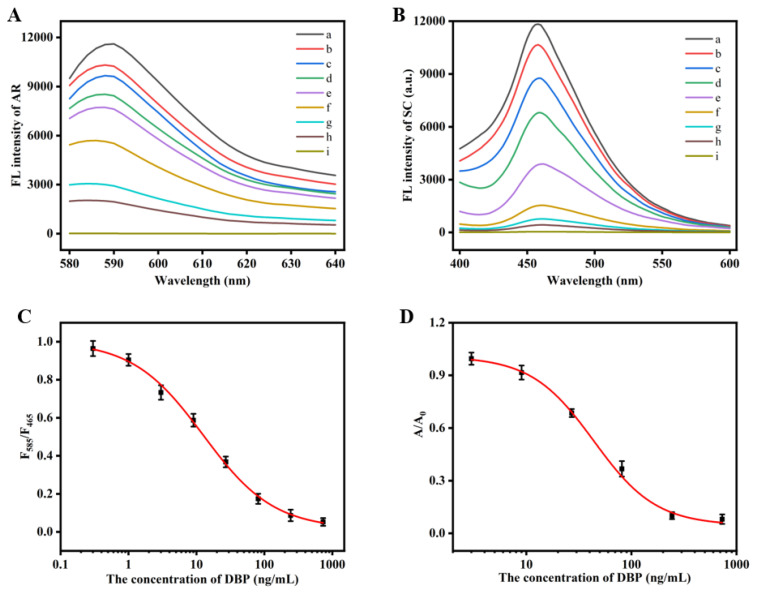
Fluorescence spectra of AR (**A**) and Sc (**B**) in the presence of increasing DBP concentrations. (**C**) Calibration curve of ratiometric fluorescence values (F_465_/F_585_) versus different concentrations of DBP (The error bars are obtained via three replicates) and (**D**) the standard curve of traditional indirect competitive ELISA for DBP under optimized conditions.

Under the above optimized parameters, the fluorescence intensity of SC at 465 nm gradually decreased with the increasing of DBP concentration, while that of AR at 585 nm gradually increased (Figure 4A,B), which indicated that the fluorescence intensity ratio (F_585_/F_465_) was negatively correlated with the DBP concentration. As shown in Figure 4C, the standard curve was drawn with Origin 9.0 based on several different concentrations of DBP standard solutions and the fluorescence intensity ratio (F_585_/F_465_). In addition, the linear regression equation of (F_585_/F_465_)/(F_585_/F_465_)_0_ = 0.01388 + 0.98997/{1 + (x/12.42809)^0.82405^} was obtained. Based on the curve, the limit of detection (LOD, 90% inhibition) was calculated as 0.86 ng/mL, which was almost 11 times lower than that of the conventional ELISA using the same antibody (seen in Figure 4D).

### 3.4. Assay Validation and Real Sample Analysis

The established method was estimated for its accuracy and precision through a spike-recovery analysis of water samples from various sources (pure water, pond water, tap water, and river water) fortified with a variety of DBP concentrations. As shown in Table 1, the satisfactory recoveries of DBP (87.2–106.62%) were obtained with the intra-assay coefficient of variation (CV) ranging from 2.57% to 6.54% by measuring three replicates, indicating that the developed method had good accuracy and could be applied for the detection of DBP from environmental samples.

The proposed method was applied to detect potential DBP in real water samples from the Zhenjiang region, together with traditional ELISA and instrument methods to further verify its reliability. As shown in Table 2 and Table S1, both results had a relatively high degree of agreement with the developed method. Meanwhile, our ratiometric fluorescence immunoassay showed better sensitivity than conventional ELISA.

## 4. Conclusions

In summary, a ratiometric fluorescence immunoassay was established for high-throughput determination of DBP based on H_2_O_2_ etching of AgNPs. Reactive oxygen species (·OH) could be produced during etching, which played a key role in fluorescence quenching of SC and fluorescence generation of AR. Under the optimized conditions, the developed method showed good accuracy and reproducibility (recoveries, 87.20–106.62%; CV, 2.57–6.54%) with higher sensitivity (LOD, 0.86 ng/mL) than traditional ELISA. Further, we believe that this work could serve as a general platform for the detection of other substances with different antibodies.

## Figures and Tables

**Table 1:** Analysis of DBP-spiked samples using this method (*n* = 3).

Samples	Background (ng/mL)	Added (ng/mL)	Found (ng/mL)	Recovery (%)	CV ^a^ (%)
Pure water	ND ^b^	5	4.36	87.20	3.73
		15	15.31	102.07	2.57
		60	58.95	98.25	4.85
River water	10.46	5	16.14	104.40	4.97
		15	23.78	93.40	2.71
		60	71.42	101.36	3.68
Tap water	ND ^b^	5	4.83	96.60	6.54
		15	13.67	91.13	5.23
		60	59.25	98.75	3.93
Pond water	21.71	5	25.97	97.23	2.91
		15	39.14	106.62	4.75
		60	75.37	92.24	3.46

CV ^a^: The intra-assay coefficient of variation obtained from three determinations; ND ^b^: not detected.

**Table 2:** Comparison of DBP detection using our established method and the conventional ELISA (*n* = 3).

Samples	Background (ng/mL)	ELISA (ng/mL)	Our Method (ng/mL)	CV ^a^ (%)
S1	ND ^b^	ND	1.12	3.57
S2	ND	ND	ND	ND
S3	ND	ND	3.97	2.35
S4	ND	ND	ND	ND
S5	ND	23.81	24.15	7.31
S6	ND	16.57	19.62	4.68
S7	ND	ND	ND	ND
S8	ND	ND	ND	ND
S9	ND	11.21	10.53	2.14
S10	ND	ND	ND	ND
S11	ND	9.62	8.36	3.95
S12	ND	19.87	20.65	2.93
S13	ND	ND	ND	ND
S14	ND	ND	ND	ND
S15	ND	ND	ND	ND

CV ^a^: Intra-assay coefficient of variation obtained from 3 determinations performed in same polystyrene microtiter plate; ND ^b^: not detected.

## Data Availability

All data are contained within the article.

## Supplementary Materials

The following supporting information can be downloaded at: https://www.mdpi.com/article/10.3390/bios12020125/s1, Materials and Instruments. LC-MS/MS analysis. Figure S1. The optimization of the concentration of H_2_O_2_ (A), SC (B), and AR (C); the optimization of different pH (D); and the optimization of different reaction times (E). Table S1. Comparison of the results of LC–MS/MS and our method using randomly spiked samples.
