# Supplementary material for: A Novel Enzyme-Free Ratiometric Fluorescence Immunoassay Based on Silver Nanoparticles for the Detection of Dibutyl Phthalate from Environmental Waters
Source: Biosensors (Basel). 2022 Feb 16;12(2):125. doi: 10.3390/bios12020125 (PMC8869742; doi:10.3390/bios12020125)
Supplement: Supplementary file 1 [file biosensors-12-00125-s001.zip › biosensors-1578826-supplementary.pdf]

biosensors

MDPI

Supplementary Materials:

A Novel Enzyme-Free Ratiometric Fluorescence Immunoassay Based on Silver Nanoparticles for the Detection of Dibutyl Phthalate from Environmental Waters

Hui Meng [1,‡], Nannan Yao [1,‡], Kun Zeng [1], Nuanfei Zhu [1], Yue Wang [1], Biying Zhao [2] and Zhen Zhang [1,*]

[1] School of the Environment and Safety Engineering, Jiangsu University, Zhenjiang 212013, China; menghui@ujs.edu.cn (H.M.); 2211909008@stmail.ujs.edu.cn (N.Y.); kjj80116@ujs.edu.cn (K.Z.); zhunuanfei@163.com (N.Z.); wangyue_persnl@126.com (Y.W.)
[2] International Genome Center, Jiangsu University, Zhenjiang 212013, China; zhaoby@ujs.edu.cn
* Correspondence: zhangzhen@ujs.edu.cn; Fax: +86-511-88790955
‡ Both authors contributed equally to this paper

Materials

$AgNO_3$, $NaBH_4$, sodium capsule, $NaHCO_3$, Na_2CO_3, $Na_2HPO_4{\cdot}12H_2O$, NaCl, KCl, $NaH_2PO_4{\cdot}2H_2O$, KH_2PO_4, ethyl alcohol, glycerinum, Tween-20, and dimethyl sulfoxide (DMSO) were purchased from the Aladdin Industrial Corporation (Shanghai, China). Scopoletin (SC) was purchased from Macklin Biotechnology Co., Ltd. (Shanghai, China). Amplex Red was purchased from Beyotime Biotechnology (Shanghai, China). Gelatin was obtained from Sigma-Aldrich (USA). Goat anti-mouse lgG was purchased from Bioss Biotechnology Co. Ltd. (Beijing, China). All solutions were prepared using ultra-pure water (18 MΩ cm, Milli-Q, Merck Millipore).

Buffers and solutions: The buffer for peroxidase-like activity substrates was 7.16 g Na_2HPO_4 and 2.1 g citric acid in 100 mL ultrapure water; the washing buffer was phosphate-buffered saline (PBS) (0.01 mol/L phosphate, pH 7.4) with 0.05% Tween-20; the carbonate buffer solution (CBS) was 1.59 g Na_2CO_3 and 2.94 g $NaHCO_3$ in 1.0 L ultrapure water and stored at 4 °C; and the blocking buffer was 1% gelatin in the CBS solution.

Instruments

The 96-well microplates were purchased from NUNC (Denmark). All chemicals were analytical grade and glass containers were cleaned by ultra-pure water before use. A transmission electron microscope (TEM) (Tecnai 12, Philips, the Netherlands), a UV−Vis spectrophotometer (UV-2600, SHIMADZU, Japan), an LC−MS/MS (1200 series and Agilent 6410 Triple Quadrupole, Agilent, Santa Clara, CA, USA), and a microplate reader (Infinite M1000 Pro, TECAN, Switzerland) were used for the measurement.

LC-−MS/MS analysis

The samples were extracted with solvent (pentane and acetone), filtered, and injected into a high-performance liquid chromatography system under constant volume conditions. The mobile phases were 5 mM ammonium acetate in water (A) and pure methanol (B). The run time between injections was 17 min. The gradient elution parameters were set as follows: 25% B, 0 s; 50% B, 120 s; 50% B, 252 s; 80% B, 258 s; 80% B, 570 s; 100% B ,576 s; and 100% B, 1020 s. The mobile phase flow rate was 0.3 mL/min and the injection volume was set as 10 μL.

Determination of DBP was completed by electrospray ionization in the positive mode with spray ion voltage (4000 V). The cone gas flow was set as 10L/min. The temperature was 350 °C and the nebulizer pressure was 276 kPa.

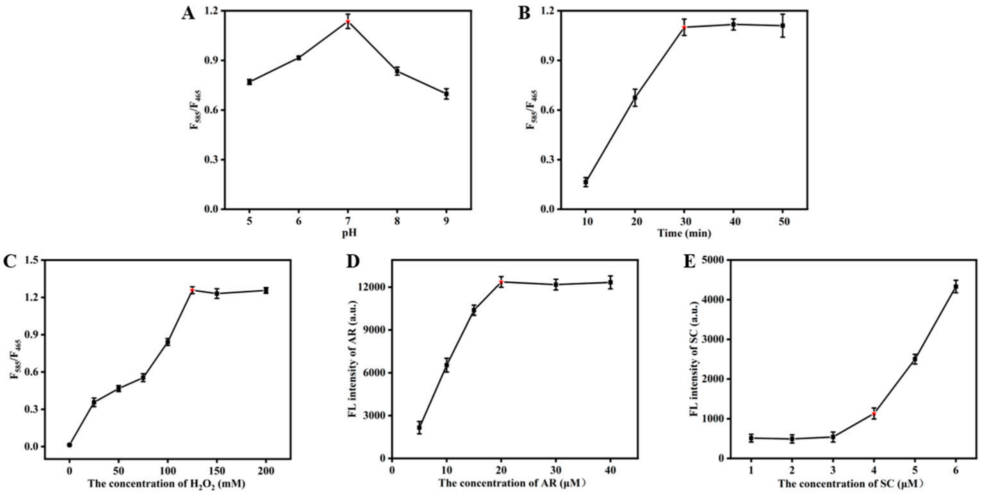

Figure S1. The optimization of the concentration of H_2O_2 (**A**), SC (**B**), and AR (**C**); the optimization of different pH (**D**); and the optimization of different reaction times (**E**).

Table S1. Comparison of the results from LC−MS/MS and our method using randomly spiked samples.

Sample	LC−MS/MS (ng/mL)	Our Method (ng/mL)
S1	8.07	8.36
S2	20.35	19.62
S3	10.84	10.53
